# Development and analytical evaluation of a microneutralization cytopathic effect assay for human adenovirus type 55-specific neutralizing antibodies

**DOI:** 10.71150/jm.2604007

**Published:** 2026-07-06

**Authors:** Dae-Im Jung, Yunjeong Park, Jung-ah Choi, Soon-Hwan Kwon, Jun Young Lee, Manki Song, Sang Hwan Seo

**Affiliations:** 1 Science Unit, International Vaccine Institute, Seoul 08826, Republic of Korea; 2 Department of Infectious Diseases, Armed Forces Medical Research Institute, Daejeon 34059, Republic of Korea; 3 KeyPrime Research, Cheongju 28161, Republic of Korea

**Keywords:** human adenovirus type 55, neutralizing antibody, plaque reduction neutralization test, microneutralization, cytopathic effect

## Abstract

Reliable quantification of neutralizing antibodies (nAb) against human adenovirus type 55 (HAdV-55) is critical for the evaluation of emerging vaccine candidates. While the plaque reduction neutralization test (PRNT) is currently the reference standard, its utility for large-scale studies is limited by low throughput, labor-intensive plaque counting, and prolonged assay times. In this study, we established and analytically validated a microneutralization assay based on cytopathic effect (MN-CPE) as a scalable alternative for HAdV-55-specific nAb quantification. Comparative performance analysis revealed that both assays maintain high dilution linearity, with coefficients of determination (R^2^) of 0.988 for MN-CPE and 0.9926 for PRNT. Relative accuracy assessments using high-, middle-, and low-titer reference sera demonstrated acceptable responses across the dynamic range. Notably, the MN-CPE assay allowed for the definition of a negative-control acceptance range, providing a distinct statistical advantage over PRNT, where negative-control values were consistently zero. Furthermore, both assays successfully detected HAdV-55-specific nAbs in immunized cynomolgus macaques, with no cross-reactivity observed against other HAdV types such as HAdV-4. These findings indicate that the MN-CPE assay is analytically comparable to PRNT and serves as a practical, relatively high-capacity alternative for HAdV-55 neutralization testing in clinical and preclinical vaccine research.

## Introduction

Human adenoviruses (HAdVs), which are non-enveloped, double-stranded DNA viruses within the *Mastadenovirus* genus, comprise over 116 genotypes categorized into seven distinct species (A–G) (Barrero et al., 2012; Lynch and Kajon, 2021). While these viruses typically cause a range of conditions—from respiratory and gastrointestinal issues to conjunctivitis—they pose a significant fatality risk to vulnerable groups like infants and the immunocompromised (Cao et al., 2014; Dodge et al., 2021; Kim and Kwon, 2024; Zhu et al., 2021). Of particular concern is HAdV-55, a potent recombinant strain of species B formed by the hexon gene exchange between HAdV-11 and HAdV-14 (Walsh et al., 2010). Since its 1969 discovery, HAdV-55 has been responsible for global outbreaks in high-density settings such as military barracks and schools (Erdman and Hierholzer, 1997; Gao et al., 2018; Hang et al., 2020; Kim and Kwon, 2024; Ko and Kwon, 2021; Liu et al., 2023; Lu et al., 2014; Walsh et al., 2010; Yoo et al., 2017; Zheng et al., 2017). Notably, this specific genotype can trigger severe pneumonia and rapid respiratory collapse even in previously healthy adults, marking it as a critical emerging public health threat (Cao et al., 2014; Gu et al., 2016; ICH, 2005; Lynch and Kajon, 2021; Tan et al., 2016; Zhang et al., 2016, 2022).

Currently, the only licensed adenovirus vaccine is an enteric-coated oral live vaccine containing wild-type HAdV-4 and HAdV-7, approved exclusively for the U.S. military (Hoke and Snyder, 2013; Lyons et al., 2008; Radin et al., 2014). While this vaccine has successfully reduced the incidence of the specific adenovirus infections, it offers limited cross-protective immunity against non-vaccine serotypes. Previous surveillance has demonstrated that outbreaks of emerging strains can persist in vaccinated populations, indicating that HAdV-4/7 immunization does not provide broad-spectrum protection against all pathogenic adenoviruses (Trei et al., 2010). To address this, efforts to establish evaluation models for vaccine development are currently underway, including the recent establishment of a cynomolgus macaque model for HAdV-55-induced respiratory disease (Seo, 2025).

In the context of HAdV-55 vaccine development, the induction of nAbs represents the most critical correlate of protection (CoP). nAbs directly inhibit viral attachment and entry into host cells, serving as a primary indicator of vaccine-induced immune protection (Cheedarla and Hanna, 2019; Pradenas et al., 2022). Therefore, accurate and reliable quantification of nAb responses is essential for evaluating vaccine immunogenicity.

The plaque reduction neutralization test (PRNT) has long been considered as the gold standard for measuring HAdV-specific nAbs (Erdman and Hierholzer, 1997; Malasig et al., 2001; Park et al., 2020). Despite its high specificity and biological relevance, the PRNT is labor-intensive, time-consuming, and inherently low throughput, with subjective plaque enumeration further limiting its suitability for analyzing large numbers of clinical samples (Denani, 2025). To address these limitations, the micro-neutralization cytopathic effect (MN-CPE) assay has been proposed as a scalable alternative, offering a more rapid approach by quantitatively assessing virus-induced cytopathic effects (Malasig et al., 2001; Manenti et al., 2020).

Here, we compared the established MN-CPE assay for HAdV-55 with PRNT in terms of dilution linearity, relative accuracy, precision, lower limit of quantification, and specificity using reference sera and cynomolgus macaque sera. The results showed that the MN-CPE assay provides a simplified and reliable platform for the assessment of HAdV-55-specific nAb titers.

## Materials and Methods

### Cells

The Vero cell line (CLB-VERO01WCB-073) was obtained from the Ministry of Food and Drug Safety (MFDS, Korea), and the A549 human lung epithelial cell line (KCLB 10185) was obtained from the Korean Cell Line Bank (KCLB, Korea). Cells were cultured in Minimum Essential Medium (MEM; Gibco, USA) supplemented with 10% heat-inactivated fetal bovine serum (FBS; Gibco) and 100 U/ml penicillin-streptomycin (Gibco) at 37°C in a humidified incubator with 5% CO₂. Subculturing was performed every 3–4 days to maintain cells in the exponential growth phase.

### Reference serum and human normal serum

Reference serum for HAdV-55 was prepared from convalescent serum collected from patients with confirmed HAdV-55 respiratory infections at the Armed Forces Capital Hospital (Korea, IRB approval No. AFMC-202208-BR-042-01). All serum samples were heat-inactivated at 56°C for 30 min prior to use to eliminate non-specific reactions. For assay qualification and data normalization, normal human serum (Serion, Germany; cat. YN058) was included in parallel during the analysis of negative (NC; cell-only) and positive (PC; virus-only) controls. Neutralization activity was calculated relative to the corresponding normal human serum values to correct for baseline interference and ensure analytical precision.

### Virus culture, purification, and titration

For virus propagation, A549 cells were seeded in T-175 flasks (Thermo Fisher Scientific, USA) at a density of 1 × 10^7^ cells/flask and incubated for 24 h. Cells were infected with HAdV-55 at a multiplicity of infection (MOI) of 2.5 in serum-free Opti-MEM (Gibco) containing 1% penicillin–streptomycin. Upon observation of approximately 70% cytopathic effect (CPE), culture supernatants were harvested and clarified by centrifugation. The supernatants were filtered through a 0.45 µm bottle-top filter (Corning, USA) and stored at -80°C. Virus purification was performed using an ÄKTA^TM^ Pure chromatography system (Cytiva, USA) equipped with a Capto^TM^ Core 700 resin column (Cytiva). The column was equilibrated with phosphate-buffered saline (PBS), and the filtered virus culture was loaded. Fractions were collected based on UV absorbance at 280 nm, concentrated using Amicon^®^ Ultra-15 centrifugal filter units (Merck Millipore, USA), and stored at -80°C. Virus titers were determined by plaque assay. A549 cells were seeded into 12-well plates (NUNC, Denmark) at 2.5 × 10^5^ cells/well and incubated overnight to form a confluent monolayer. Virus samples were serially diluted tenfold, and 200 µl of each dilution was inoculated. After a 2 h adsorption at 37°C, the inoculum was removed, and cells were overlaid with MEM containing 2% FBS and 0.8% agarose. After 7 days of incubation at 37°C, cells were fixed with 4% paraformaldehyde (Hanlab, Korea) and stained with 2.3% crystal violet (Sigma-Aldrich, USA). Viral titers were calculated as plaque-forming units (PFU)/ml using the following formula:


$$\text{PFU/ml }=\;\frac{\text{number of plaques per well}\times \text{dilution factor}}{\text{inoculum volume (ml)}}$$

### Microneutralization Cytopathic effect (MN-CPE) assay

Reference serum was diluted 1:10 in serum-free MEM and further subjected to serial twofold dilutions. In a 96-well plate, 5 µl of diluted serum was mixed with 45 µl of virus suspension containing 150 PFU. NC and PC wells received 5 µl of normal human serum. Serum–virus mixtures were incubated at 37°C for 1 h for neutralization, followed by static incubation at 4°C for 16–20 h, which allows trace neutralizing antibodies in low-titer clinical specimens to fully bind virus particles in the minimal volumes used in 96-well format, thereby enhancing assay sensitivity for vaccine trial samples. Plates were equilibrated to room temperature (20–24°C) for 30 min prior to cell seeding. A549 cells were added at a density of 1.5 × 10^4^ cells/well in MEM with 2% FBS, and plates were incubated at 37°C with 5% CO_2_ for 5 days. After incubation, cells were washed with PBS, fixed with 4% paraformaldehyde for 2 h, and stained with 2.3% crystal violet. Absorbance was measured at 570 nm using a microplate reader (SpectraMax ABS Plus; Molecular Devices, USA). The nAb activity was expressed as the neutralization rate (%), calculated relative to the optical density (O.D.) of the NC. Samples with ≥ 30% neutralization were considered positive, as samples showing < 30% neutralization consistently exhibited complete virus-induced CPE during assay optimization using reference sera. The highest serum dilution meeting 30% neutralization was defined as the nAb titer (IC_30_: 30% inhibition concentration).


$$\mathrm{neutralization}\;\mathrm{rate}\;\left(\%\right)=\frac{O.D.\;\mathrm{sample}}{O.D.\;\mathrm{negative}\;\mathrm{control}}\times 100$$

### Plaque reduction neutralization test (PRNT)

A549 cells were seeded into 12-well plates at 2.5 × 10^5^ cells/well and incubated for 24 h. Serum samples were initially diluted 1:20 and subjected to serial twofold dilutions. Each dilution was mixed 1:1 (v/v) with HAdV-55 (100 PFU/100 µl) and incubated at 37°C for 60 min. NC and PC wells received 1:20 diluted normal human serum. Mixtures were inoculated onto cell monolayers for 1 h at 37°C. After removing the inoculum, cells were overlaid with 1 ml of MEM containing 2% FBS and 0.8% agarose (Lonza, Switzerland) and incubated for 7 days. Following fixation and staining, plaques were manually counted. The nAb titers were expressed as the reciprocal of the highest serum dilution reducing plaque counts by 50% (PRNT_50_). PRNT_50_ values were derived using a four-parameter logistic (4-PL) regression model.

### Qualification of neutralization assays and data analysis

Qualification of PRNT and MN-CPE assays was performed in accordance with ICH Q2(R1) (ICH, 2005) guidelines to assess linearity, accuracy, precision (repeatability and intermediate precision), LOD, LLOQ, and acceptance ranges for control samples. Reference sera were prepared at high (undiluted), Middle (0.5-fold), and Low (0.1-fold) concentrations using normal human serum as the diluent.

Linearity was evaluated by analyzing reference serum prepared through serial two-fold dilutions. Observed nAb titers were plotted against the dilution factors, and the assessment was performed using linear regression analysis. The acceptance criteria were defined as a coefficient of determination (R^2^) ≥ 0.9.

Accuracy was assessed using high, middle, and low nAb concentration reference serum, with each sample further diluted at levels ranging from 1:1.25 to 1:5. Relative accuracy was determined by calculating the percent recovery, defined as the ratio of observed to expected values. The acceptable recovery range was established at 100 ± 50%.


Expected value = [dilution factor × observed value]

$$\text{\% recovery }=\;\frac{\text{observed value}}{\text{expected value}}\;\times \;100$$

Precision was evaluated through repeatability (intra-plate and inter-plate) and intermediate precision (between operators and days). The acceptance criterion for all precision parameters was a coefficient of variation (CV) < 60%.


$$\text{CV (\%) }=\;\frac{\text{standard deviation of nAb titer}}{\text{Geometric mean of nAb titer}}\;\times \;100$$

The LOD was determined based on the distributional characteristics of the raw data. Prior to calculation, the intra-quintuplicate precision of the data was verified to ensure that the %CV was ≤ 40%. The normality of the data set was assessed to select the appropriate calculation method. For data following a normal distribution, the LOD was calculated using the following formula: LOD = Mean + 1.645 × standard deviation (SD). This value corresponds to the 95th percentile upper distribution limit. In cases where the raw data did not meet the normality assumption, a log transformation was applied. If the transformed data achieved normality, the LOD was calculated by determining the "transformed Mean + 1.645 × transformed SD" and subsequently re-transforming the result. If normality was still not achieved after log transformation, the LOD was determined using the 95% value among the samples. The LLOQ was derived from the calculated LOD using the formula: LLOQ = LOD × 3.3.

Acceptance ranges for NC were established using a similar statistical approach. Ranges were determined using the mean and *t*-threshold at a 95% confidence interval (n-1 degrees of freedom). For two-sided limits, the *t*-threshold was calculated as TINV (0.05, n-1), while single-sided limits were adjusted using TINV (0.05 × 2, n-1). Data were analyzed as Mean ± (*t*-threshold × standard deviation) for normal distributions, or back-transformed after log-analysis for non-normal data.

### Preparation of vaccine candidate (virus inactivation, test inactivation, AH mixing, and unit calculation)

For antigen production, Vero cells were seeded into 10-layer CellStack^TM^ culture chambers (Corning) at 5 × 10^7^ cells/chamber and incubated for 24 h. Cells were inoculated with HAdV-55 at an MOI of 5 and cultured in Opti-MEM for 3 days. Viral antigens were harvested via two freeze–thaw cycles at -70°C, followed by centrifugation to remove cell debris. Supernatants were sterile-filtered through a 0.2 µm Sartopore^®^ 2 MidiCap filter (Sartorius, Germany) and stored at -70°C prior to purification.

Purification was performed using Capto^TM^ Core 700 resin-based flow-through chromatography as previously described. Virus-containing fractions were collected based on UV absorbance at 280 nm. For virus inactivation, 0.1 M formaldehyde was added to the chromatography pool, followed by incubation at 37°C for 24 h with continuous agitation. Residual formaldehyde was removed by ultrafiltration and diafiltration (UF/DF) using a 300 kDa molecular weight cut-off membrane (Sartorius) with PBS buffer exchange.

The final vaccine formulation was adjusted to an antigen concentration of 1,000 IU/ml with 0.5 mg/ml aluminum hydroxide gel (Sigma-Aldrich) in PBS. The formulation was aseptically filled into vials and stored at 2–8°C until use.

### Animal care and housing conditions

Three male cynomolgus macaques (*Macaca fascicularis*; 3–4 years old) were acclimated for at least one week prior to HAdV-55 vaccine candidate inoculation. Animals were maintained individually in stainless-steel cages (720 × 700 × 800 mm) in a BSL-2 animal facility under controlled conditions of 25 ± 1°C, 50 ± 10% relative humidity, and a 12-h light/dark cycle. Monkeys received a commercial primate diet twice daily and had unrestricted access to UV-irradiated, filtered water. Each animal was individually identified and housed separately to reduce the potential for cross-contamination.

### Ethics and biosafety statement

All procedures involving animals were conducted following the *Guide for the Care and Use of Laboratory Animals* (8th ed., ILAR) and the Animal Welfare Act. The experiments were carried out at Keyprime Research (KPR), an AAALAC-accredited facility, under protocols approved by the KPR Institutional Animal Care and Use Committee (IACUC-AP-25-024). Cynomolgus monkeys imported from Nafovanny (Vietnam) underwent a minimum 30-day quarantine in compliance with Republic of Korea APQA regulations and were screened for major simian pathogens. A veterinarian verified the health status of all animals before study initiation. All procedures involving infectious HAdV-55 were performed in a BSL-2 facility, and waste materials were autoclaved prior to disposal. Environmental enrichment was provided according to institutional policies.

### Study design

All cynomolgus macaques were verified to be seronegative for HAdV-55-neutralizing antibodies before immunization (data not shown). Three animals received intramuscular injections of 250 IU of the inactivated HAdV-55 formulated with aluminum hydroxide into the left rectus femoris. Immunizations were administered twice, with a 4-week interval between doses (see Fig. 5A). Blood samples were collected on days 0, 28, and 56, then processed to obtain serum and stored at -80°C until subsequent analysis for MN-CPE and PRNT assays.

## Results

### Dilution linearity of MN-CPE and PRNT

While the MN-CPE assay has been established for measuring virus-specific nAbs, its validation for HAdV-55 nAb detection and direct comparison with PRNT have not been reported. Thus, we evaluated and compared the dilution linearity of MN-CPE and PRNT to determine their analytical performance and consistency in quantifying HAdV-55-specific nAbs. To assess the analytical performance of the MN-CPE assay for HAdV-55, we first examined dilution linearity using serial dilutions of a reference human serum and compared the results with those obtained by PRNT. In both assays, the measured neutralizing antibody titers showed a strong linear relationship with the diluted reference serum across the tested range (Fig. 1). The MN-CPE assay demonstrated excellent dilution linearity, with a coefficient of determination of R^2^ = 0.988 (Fig. 1A). Similarly, PRNT also exhibited strong linearity, with an R^2^ value of 0.9926 (Fig. 1B). These results indicate that the MN-CPE assay performs comparably to PRNT in terms of dilution-dependent response and supports its suitability for quantifying HAdV-55-specific neutralizing antibody activity.

### Accuracy of MN-CPE and PRNT assays

To further assess the analytical performance of the MN-CPE assay, we evaluated its linearity and relative accuracy using serial dilutions of high-, middle-, and low-titer reference sera and compared the results with those obtained by PRNT (Fig. 2). In the MN-CPE assay, the observed HAdV-55-specific nAb titers showed a strong linear relationship with the expected titers across all three reference sera, with coefficients of determination (R^2^) of 0.9717, 0.988, and 0.9681 for the high-, middle-, and low-titer samples, respectively (Fig. 2A). Similarly, PRNT also demonstrated strong linearity, with R^2^ values of 0.9947, 0.9897, and 0.9786 for the corresponding high-, middle-, and low-titer reference sera (Fig. 2B).

Relative accuracy was then assessed by calculating recovery rates for each dilution. In the MN-CPE assay, the mean recovery rates were 101.63% for the high-titer serum, 83.01% for the middle-titer serum, and 100.91% for the low-titer serum (Table S1). In PRNT, the corresponding mean recovery rates were 113.09%, 110.24%, and 118.87%, respectively (Table S2). Overall, both assays showed good linearity over the tested concentration range, whereas recovery rates were more consistent for the high-titer reference serum and showed greater deviation in the middle- and low-titer ranges. These findings indicate that the MN-CPE assay has analytical performance comparable to PRNT in terms of dilution-dependent response and relative accuracy, supporting its applicability for quantifying HAdV-55-specific nAb responses.

### Precision (Repeatability and intermediate precision) of MN-CPE and PRNT

To evaluate assay precision, variability was assessed at the intraplate, interplate, and intermediate levels for both MN-CPE and PRNT using high-, middle-, and low-titer reference sera (Fig. 3). The MN-CPE assay showed average coefficients of variation (CVs) of 28.97% for intraplate precision, 35.04% for interplate precision, and 37.94% for intermediate precision. In contrast, PRNT showed consistently lower variability, with average CVs of 12.01%, 7.81%, and 6.76% for intraplate, interplate, and intermediate precision, respectively. This difference in precision is likely attributable to the distinct approaches used to determine neutralization titers in the two assays. PRNT calculates inhibitory concentrations using a 4-parameter logistic (4PL) regression model, enabling a more continuous estimation of titers and thereby reducing variability. In contrast, MN-CPE determines titers based on discrete serum dilution steps, which inherently results in larger numerical increments and greater variation between measurements. Nevertheless, both assays showed consistent performance within their respective analytical frameworks, indicating that MN-CPE as well as PRNT is reliable for measuring HAdV-55-specific nAb titers within the context of its intended use.

### Lower limit of quantification (LLOQ) of MN-CPE and PRNT

To determine the lower limit of quantification (LLOQ), diluted reference sera were tested repeatedly and the mean titer and variability at each dilution were assessed for both MN-CPE and PRNT (Fig. 4). In the MN-CPE assay, the LLOQ was established at 26.39 IC_30_, corresponding to diluted reference serum B, whereas the PRNT assay showed an LLOQ of 21.46 nAU/ml at the same dilution level. Below these values, quantification was unreliable, as reflected by the high variability observed in the lowest dilution point of MN-CPE and the non-quantifiable range in PRNT. Across the quantifiable range, MN-CPE produced mean observed titers from 26.39 to 735.17 IC_30_, while PRNT yielded titers from 21.46 to 576.66 nAU/ml. Collectively, these results support that MN-CPE has analytical sensitivity comparable to PRNT for the quantification of HAdV-55-specific nAbs.

### Acceptance range of negative control for MN-CPE and PRNT

To establish assay acceptance criteria, negative control values were analyzed for both MN-CPE and PRNT (Tables 1 and 2). For MN-CPE, the distribution of raw negative control O.D. values did not satisfy the normality assumption. However, after log transformation, the data showed an acceptable normal distribution, allowing the acceptance range to be determined on the log scale and then back-transformed. Based on this analysis, the acceptance range for the MN-CPE negative control was defined as 0.954 to 1.302 O.D., with a log mean of 0.047 and a log standard deviation of 0.033 (Table 1). In contrast, all negative control values in PRNT were 0 across the 30 tested samples, resulting in both the upper and lower acceptance limits being set to 0 (Table 2). Collectively, these results define the negative control acceptance criteria for both assays. While PRNT demonstrated a highly stringent baseline response, MN-CPE established a reproducible acceptance range for negative controls, providing a practical framework for assay standardization and quality control. These findings support the applicability of MN-CPE in routine testing settings and relatively higher sample processing capacity compared than the conventional PRNT assay.

### Detection of vaccine-induced HAdV-55-specific nAb

To evaluate assay specificity and potential cross-neutralization in a biologically relevant setting, sera from cynomolgus macaques, which are commonly exposed to diverse simian adenoviruses, were analyzed using both MN-CPE and PRNT assays. Specificity was evaluated using sera collected from cynomolgus macaques immunized twice with inactivated HAdV-55 plus aluminum hydroxide (AH) and tested against HAdV-55 and HAdV-4 by MN-CPE and PRNT (Fig. 5A). In both assays, HAdV-4-specific nAb responses were undetectable or negligible at all time points, whereas HAdV-55-specific nAb titers were consistently detected following immunization and increased progressively through day 56 (Fig. 5B and 5C). Representative MN-CPE and PRNT images confirmed these findings, showing inhibition of cytopathic effect or plaque formation only in the homologous HAdV-55 assay, but not in the heterologous HAdV-4 assay (Figs. 5D, 5E and S1). These results indicate that MN-CPE, like PRNT, specifically measures HAdV-55-nAb responses with minimal cross-reactivity.

## Discussion

In the present study, we established and analytically evaluated an MN-CPE assay for the measurement of HAdV-55-specific nAbs and compared its performance with that of the conventional PRNT. PRNT has been widely used as a reference method for neutralizing antibody measurement, but its practical limitations, including low throughput, prolonged assay time, and labor-intensive plaque visualization and counting, restrict its utility in large-scale studies and routine testing settings (Fries et al., 2025; Goh et al., 2016). In this context, microneutralization-based approaches have been explored as practical alternatives for neutralization testing, including adenovirus assays using colorimetric or reporter-based readouts (Crawford-Miksza and Schnurr, 1994; Li and Wadell, 1986; Malasig et al., 2001). Our results showed that the MN-CPE assay was broadly comparable to PRNT with respect to dilution linearity, relative accuracy, analytical sensitivity, and serotype specificity, while also providing an absorbance-based assay format that may facilitate standardization and quality control.

Both MN-CPE and PRNT showed strong dilution-dependent responses in the linearity analysis. The coefficients of determination were 0.988 for MN-CPE and 0.9926 for PRNT, indicating close agreement between the two assays over the tested range. In addition, when high-, middle-, and low-titer reference sera were evaluated, both methods maintained acceptable linearity and recovery across serial dilutions. These findings indicate that the MN-CPE assay can provide quantitative measurements comparable to those obtained with PRNT. Similar validation studies of microneutralization assays for other viral systems have also shown that the neutralization formats can achieve acceptable linearity, accuracy, precision, and specificity when properly qualified (Fries et al., 2025).

In the precision analysis, the MN-CPE assay showed higher variability than PRNT. The average CV values for MN-CPE were 28.97%, 35.04%, and 37.94% for intraplate, interplate, and intermediate precision, respectively, whereas the corresponding values for PRNT were 12.01%, 7.81%, and 6.76%. This difference is likely attributable to the distinct methods used for endpoint determination. PRNT derives inhibitory concentrations from regression-based analysis, allowing a more continuous estimation of titer values, whereas MN-CPE determines titers on the basis of discrete dilution steps. Accordingly, somewhat greater numerical variation is expected in the MN-CPE format. However, despite this difference, the MN-CPE assay retained strong linearity and acceptable relative accuracy, indicating that its analytical performance remained suitable for neutralization measurement within the tested range.

The notable distinction between the two assays was observed in the negative-control analysis. In PRNT, all negative-control values were zero, resulting in an acceptance range of 0–0. Although this reflects a stringent baseline response, it does not provide a measurable distribution for monitoring run-to-run background variation. In contrast, MN-CPE generated a definable negative-control range after log transformation (O.D. 0.954–1.302), which may provide a practical basis for assay control and routine quality monitoring. This interpretation is consistent with general analytical validation principles emphasizing assay suitability, reportable range, precision, and system suitability for the intended purpose, and with recent microneutralization validation studies that incorporated predefined internal quality control ranges for routine assay monitoring (Fries et al., 2025).

The specificity study using sera from immunized cynomolgus macaques further supported the utility of the MN-CPE assay. In both MN-CPE and PRNT, sera collected after immunization showed increased neutralizing activity against HAdV-55, whereas responses against the heterologous serotype HAdV-4 remained negligible. Representative plate images also showed inhibition only in the homologous HAdV-55 assay. These findings indicate that the MN-CPE assay can specifically detect HAdV-55-neutralizing antibodies with minimal cross-reactivity under the conditions tested. This is biologically plausible because adenovirus neutralizing antibody responses are largely serotype-specific, with hexon hypervariable regions serving as major determinants of type-specific neutralization; HAdV-55 and HAdV-4 likewise have distinct neutralizing epitopes and limited serologic cross-reactivity (Feng et al., 2018; Tian et al., 2015, 2018).

For early-stage assay qualification, representative high-, middle-, and low-response samples were used to assess analytical performance in a fit-for-purpose manner, consistent with ICH Q2(R2) and FDA guidance (FDA, 2018; ICH, 2023). Due to limited access to independent high-, middle-, and low-titer human serum samples, these materials were generated by serial dilution of a single positive reference serum using HAdV-55-seronegative human serum as the dilution matrix. However, this approach may not fully reflect inter-individual variability in human neutralizing antibody responses. Therefore, broader validation using a larger panel of independent human samples will be necessary before application to phase 3 clinical sample analysis.

Taken together, the present results indicate that the MN-CPE assay has analytical characteristics comparable to those of PRNT for the measurement of HAdV-55-specific neutralizing antibodies. Although MN-CPE showed higher variability than PRNT, it demonstrated acceptable linearity, relative accuracy, specificity, and a practical framework for negative-control monitoring. These features suggest that MN-CPE may serve as a useful alternative to PRNT for HAdV-55 neutralization studies, particularly when a more standardized and higher-capacity assay format to conventional PRNT assay is required.

## Figures and Tables

**Fig. 1.**
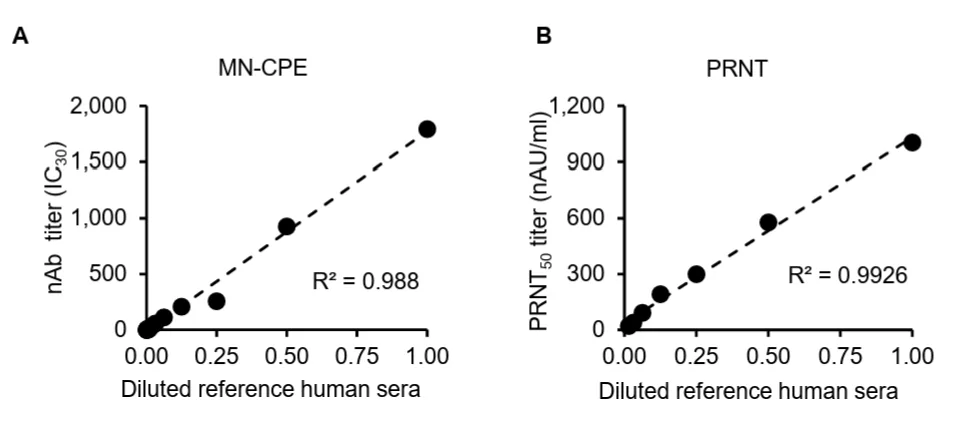
Dilution linearity analysis of MN-CPE and PRNT assays. Serial dilutions of a reference human serum were tested using the MN-CPE assay (A) and PRNT (B). nAb titers are shown as nAU/ml for MN-CPE and PRNT_50_ values (nAU/ml) for PRNT. Linear regression analysis was performed to assess dilution linearity, and the coefficient of determination (R²) is indicated in each panel. AU, arbitrary unit.

**Fig. 2.**
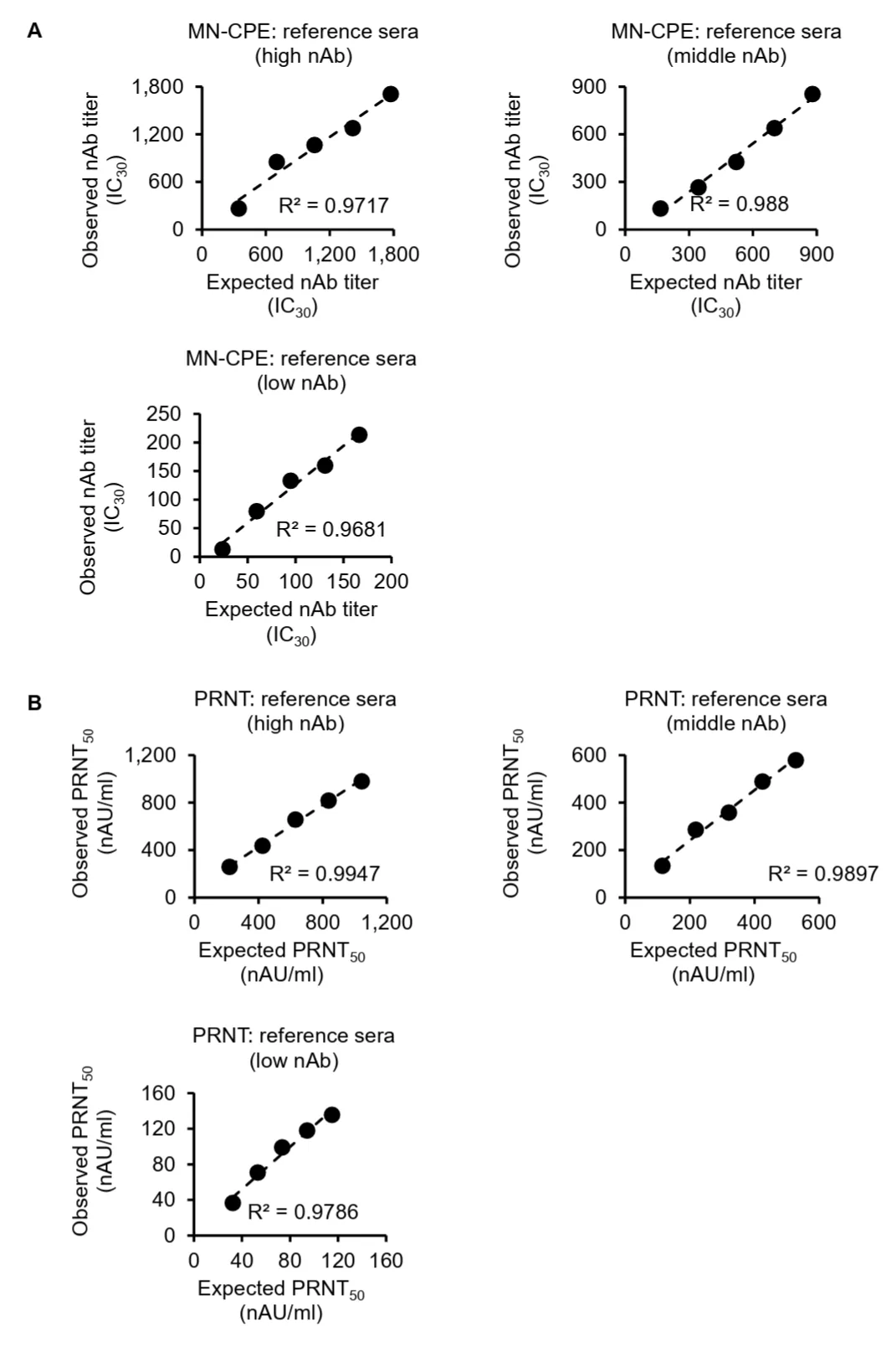
Linearity and relative accuracy of the MN-CPE and PRNT assays. High-, middle-, and low-titer reference sera were serially diluted and analyzed by MN-CPE (A) and PRNT (B). Scatter plots show the relationship between expected and observed nAb titers, and the corresponding R^2^ values are indicated. Tables summarize the expected titers, observed titers, recovery rates, and average recovery rates for each reference serum. AU, arbitrary unit; NC, not calculable; NA, not applicable.

**Fig. 3.**
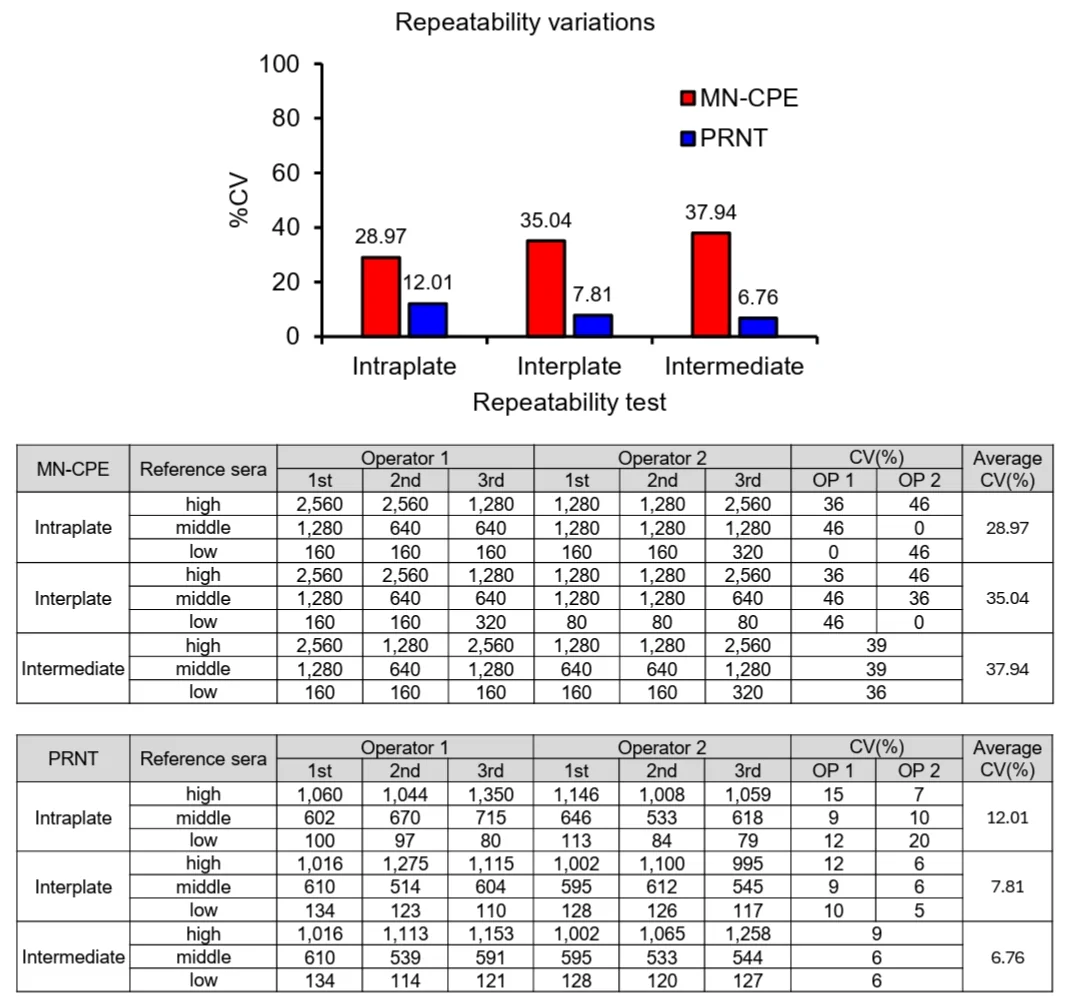
Comparison of precision between MN-CPE and PRNT assays. Precision was evaluated for MN-CPE and PRNT using high-, middle-, and low-titer reference sera. Intraplate, interplate, and intermediate precision were assessed using repeated measurements by two operators. Tables show the individual titer values and coefficients of variation (CVs), and the graph summarizes the average CV values for each assay condition. OP, operator.

**Fig. 4.**
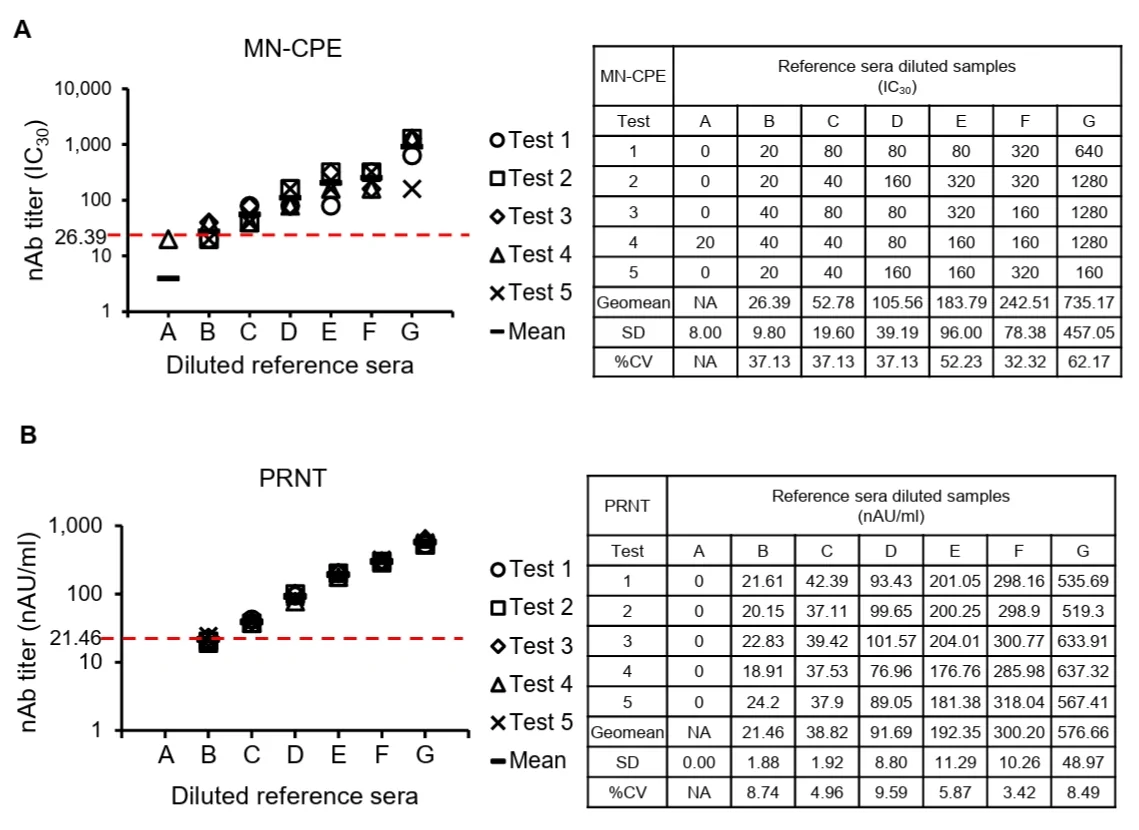
Determination of the lower limit of quantification (LLOQ) for MN-CPE and PRNT assays. Diluted reference sera were repeatedly tested by MN-CPE (A) and PRNT (B) to determine the LLOQ. Tables show the measured nAb titers for each test, together with the mean, standard deviation, and %CV values. The lowest concentration that met the predefined quantification criterion was defined as the LLOQ. AU, arbitrary unit; NA, not applicable.

**Fig. 5.**
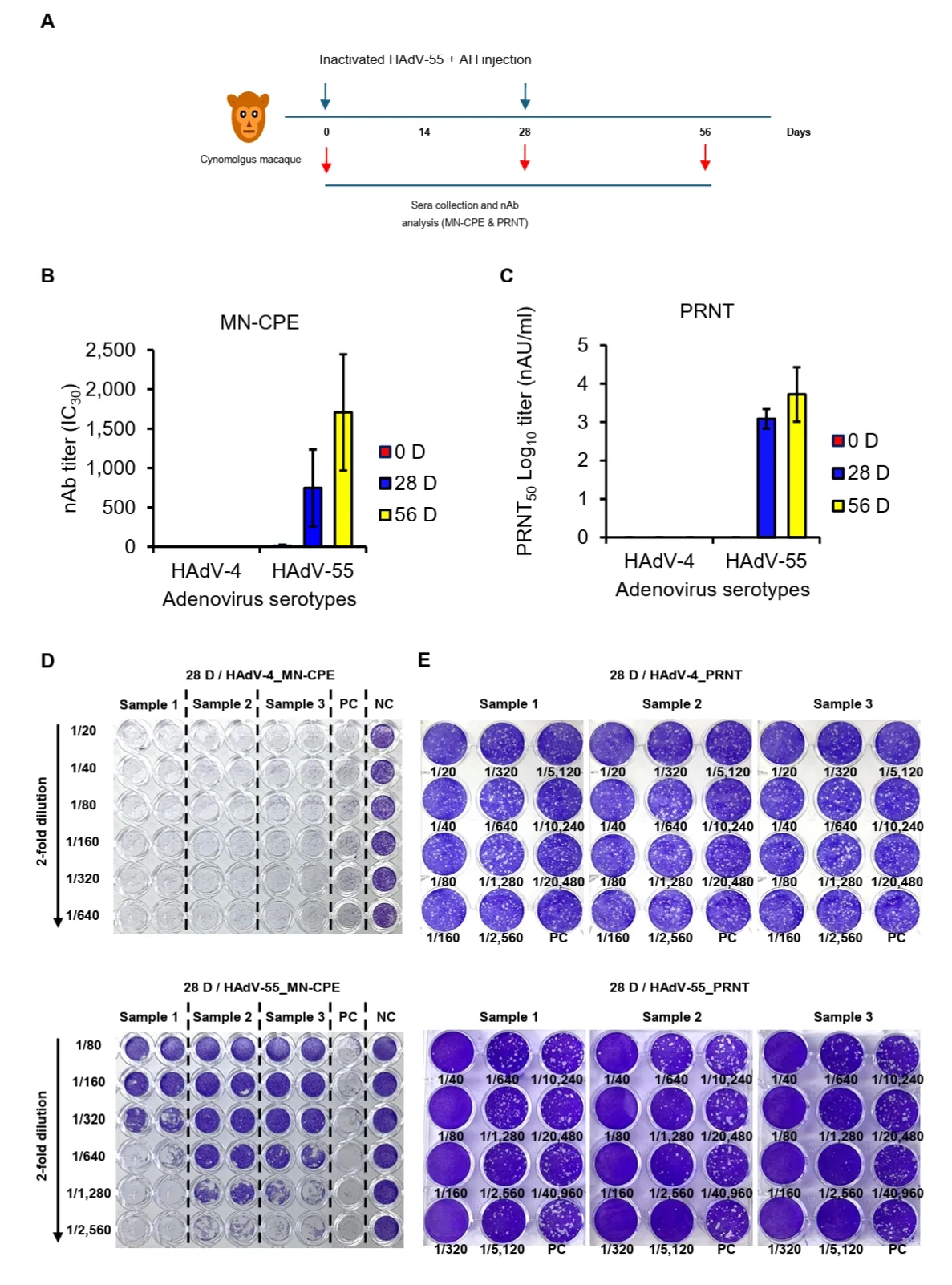
Specificity of HAdV-55-specific responses in MN-CPE and PRNT assays. (A) Experimental schedule for immunization of cynomolgus macaques with inactivated HAdV-55 formulated with aluminum hydroxide and subsequent serum collection. nAb responses against HAdV-4 and HAdV-55 were measured by MN-CPE (B) and PRNT (C) at the indicated time points. Representative assay images for MN-CPE (D) and PRNT (E) using sera collected after immunization are shown. PC, positive control; NC, negative control; AU, arbitrary unit.

**Table 1.** Acceptance range of negative control for MN-CPE

Sample	O.D.	Sample	O.D.
#1	1.176	#16	1.003
#2	1.267	#17	0.983
#3	1.127	#18	1.122
#4	1.083	#19	1.149
#5	1.046	#20	1.168
#6	1.062	#21	1.132
#7	1.038	#22	1.267
#8	1.116	#23	1.010
#9	1.206	#24	1.080
#10	1.409	#25	1.125
#11	1.085	#26	1.083
#12	1.182	#27	1.077
#13	1.077	#28	1.144
#14	0.994	#29	1.093
#15	1.143	#30	1.061
Normality test			No
Normality test using log data			Yes
Log Mean			0.047
Log SD			0.033
*t*-Threshold			2.045
Log upper limit			0.114
Log lower limit			-0.020
Upper limit			1.302
Lower limit			0.954

**Table 2.** Acceptance range of negative control for PRNT

Sample	O.D.	Sample	O.D.
#1	0	#16	0
#2	0	#17	0
#3	0	#18	0
#4	0	#19	0
#5	0	#20	0
#6	0	#21	0
#7	0	#22	0
#8	0	#23	0
#9	0	#24	0
#10	0	#25	0
#11	0	#26	0
#12	0	#27	0
#13	0	#28	0
#14	0	#29	0
#15	0	#30	0
Normality test			No
Log normality test			No
Upper limit			0
Lower limit			0

## Supplementary Information

Table S1. Linearity and relative accuracy of the MN-CPE

Table S2. Linearity and relative accuracy of the PRNT

Fig. S1. Representative MN-CPE and PRNT assay images using cynomolgus macaque sera collected before and after immunization. Representative plate images of MN-CPE and PRNT assays performed using sera collected at day 0 (pre-immunization) and day 56 (post-immunization) from cynomolgus macaques (n = 3). Neutralizing activity against HAdV-4 and HAdV-55 is shown for comparison. PC, positive control; NC, negative control.
