# Supplementary material for: Development and analytical evaluation of a microneutralization cytopathic effect assay for human adenovirus type 55-specific neutralizing antibodies
Source: J Microbiol. 2026 Jul 6;64(7):e2604007. doi: 10.71150/jm.2604007 (PMC13687993; doi:10.71150/jm.2604007)
Supplement: Supplementary file 1 [file jm-2604007-Supplementary-Fig-S1.pdf]

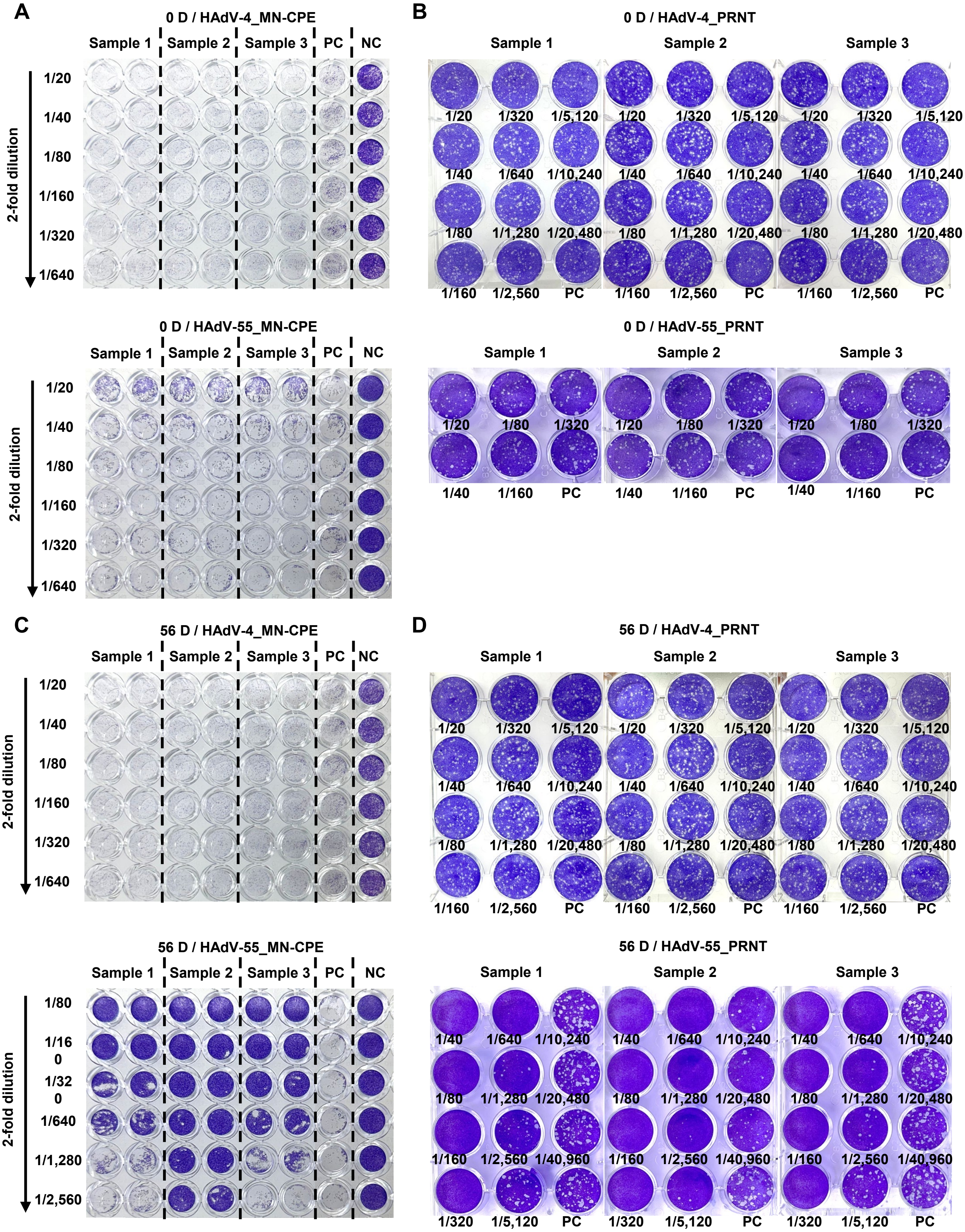

Fig. S1. Representative MN-CPE and PRNT assay images using cynomolgus macaque sera collected before and after immunization. Representative plate images of MN-CPE and PRNT assays performed using sera collected at day 0 (pre-immunization) and day 56 (post-immunization) from cynomolgus macaques (n = 3). Neutralizing activity against HAdV-4 and HAdV-55 is shown for comparison. PC, positive control; NC, negative control.
