# Supplementary material for: Development and analytical evaluation of a microneutralization cytopathic effect assay for human adenovirus type 55-specific neutralizing antibodies
Source: J Microbiol. 2026 Jul 6;64(7):e2604007. doi: 10.71150/jm.2604007 (PMC13687993; doi:10.71150/jm.2604007)
Supplement: Supplementary file 2 [file jm-2604007-Supplementary-Table-S1.pdf]

Table S1. Linearity and relative accuracy of the MN-CPE

Reference sera	Dilution	Expected nAb (IC_{30})	Observed nAb (IC_{30})	Recovery rate (%)	Average recovery rate (%)
High	1	1612.70	1612.70	100.00	101.63
	1:1.25	1290.16	1280.00	99.21	
	1:1.67	965.69	1015.94	105.20	
	1:2.5	645.08	806.35	125.00	
	1:5	322.54	253.98	78.75	
Middle	1X	806.35	806.35	100.00	83.01
	1:1.25	645.08	640.00	99.21	
	1:1.67	690.96	403.17	58.35	
	1:2.5	322.54	253.98	78.75	
	1:5	161.27	126.99	78.75	
Low	1X	201.59	201.59	100.00	100.91
	1:1.25	161.27	160.00	99.21	
	1:1.67	120.71	126.99	105.20	
	1:2.5	80.63	80.00	99.21	
	1:5	40.32	NC	NA	
