# Supplementary material for: Development and analytical evaluation of a microneutralization cytopathic effect assay for human adenovirus type 55-specific neutralizing antibodies
Source: J Microbiol. 2026 Jul 6;64(7):e2604007. doi: 10.71150/jm.2604007 (PMC13687993; doi:10.71150/jm.2604007)
Supplement: Supplementary file 3 [file jm-2604007-Supplementary-Table-S2.pdf]

Table S2. Linearity and relative accuracy of the PRNT

Reference sera	Dilution	Expected $PRNT_{50}$ (nAU/ml)	Observed $PRNT_{50}$ (nAU/ml)	Recovery rate (%)	Average recovery rate (%)
High	1	964.87	964.87	100.00	113.09
	1:1.25	771.89	814.57	105.53	
	1:1.67	577.76	656.36	113.60	
	1:2.5	385.95	434.02	112.46	
	1:5	192.97	258.28	133.84	
Middle	1X	570.10	570.10	100.00	110.24
	1:1.25	456.08	484.28	106.18	
	1:1.67	341.37	356.28	104.37	
	1:2.5	228.04	282.47	123.87	
	1:5	114.02	133.14	116.77	
Low	1X	135.56	135.56	100.00	118.87
	1:1.25	108.45	117.74	108.57	
	1:1.67	81.18	98.99	121.95	
	1:2.5	54.22	70.18	129.42	
	1:5	27.11	36.44	134.41	
